# Middle molecules in severe community-acquired pneumonia

**DOI:** 10.1186/2197-425X-3-S1-A791

**Published:** 2015-10-01

**Authors:** T Dumitras, V Garbuz, C Gutu-Bahov, S Matcovschi, I Camerzan, N Gurschi

**Affiliations:** State University of Medicine and Pharmacy 'Nicolae Testemitanu', Department of Internal Medicine, Chisinau, Republic of Moldova; State University of Medicine and Pharmacy 'Nicolae Testemitanu', Department of Anaesthesiology and Reanimatology Nr.2, Chisinau, Republic of Moldova; Sfanta Treime Municipal Hospital, Critical Care Unit, Chisinau, Republic of Moldova; Sfanta Treime Municipal Hospital, Department of Toxicology, Chisinau, Republic of Moldova

## Introduction

In recent years there is a revival of interest to the plasma level of middle molecules which is the marker of activation of endogenous proteolysis and the expressiveness of endogenous intoxication syndrome.

## Objectives

To examine the state of middle molecules (MM) in the blood plasma of patients with severe community-acquired pneumonia (CAP).

## Methods

Retrospective analysis of 23 cases of severe CAP hospitalized in Critical Care Unit of Sfanta Treime Municipal Hospital, Chisinau, from October 2014 to February 2015. The measurements of level of the peptide substances belonging to a group of “middle molecules” (MM) using spectrophotometer were carried out and the results were compared to the levels of 11 healthy controls.

## Results

Patients admitted had mean age of 62 ± 1.2 years, PORT classes IV-V and mean Pneumonia Severity Index score of 108.5 ± 3.8. In all patients on admission there was a high level of endogenous intoxication manifested by 1.8 times increasing levels of MM compared to the control group (0.262 ± 0.001 versus 0.146 ± 0.02 optic units, p < 0.05). There was a positive trend with a decline of MM on day 7 - 0.248 ± 0.001. No positive correlation was found between MM and other laboratory parameters with the exception of increased band neutrophils (r = 0.71, p < 0.05). ICU-mortality constituted 21.7% (5/23). In patients who died MM level was 0.438 ± 0.002 optic units, which was 3 times higher compared to the control group (p < 0.05).

## Conclusions

Determining the plasma level of middle molecules can be used as an additional dynamic criterion of severity of endogenous intoxication in community-acquired pneumonia.
